# Performance of the Malmgren Index for Assessing Root Resorption on 2D vs. 3D Radiographs: A Pilot Study

**DOI:** 10.3390/healthcare11131860

**Published:** 2023-06-26

**Authors:** Hanne Michielsens, Julie Decreus, Giacomo Begnoni, Anna Verdonck, Reinhilde Jacobs, Guy Willems, Maria Cadenas de Llano-Pérula

**Affiliations:** 1 Department of Oral Health Sciences-Orthodontics, KU Leuven and Dentistry, University Hospitals Leuven, 3000 Leuven, Belgium; hanne.michielsens@uzleuven.be (H.M.); julie.decreus@uzleuven.be (J.D.);; 2 OMFS IMPATH, Department of Imaging & Pathology, Faculty of Medicine, KU Leuven and Maxillofacial Surgery, University Hospitals Leuven, 3000 Leuven, Belgium; 3 Department of Dental Medicine, Karolinska Institutet, 171 77 Stockholm, Sweden

**Keywords:** panoramic radiographs, CBCT, Malmgren index, root resorption

## Abstract

Objectives: To compare the performance of the Malmgren index on 2D and 3D radiographs. Methods: Patients with a panoramic radiograph and a cone beam computed tomography (CBCT) taken at an interval of <3 months and presenting root resorption (RR) on at least one incisor and/or canine were retrospectively included. RR was scored twice by two observers using the Malmgren index in both the 2D and 3D sets, and intra-class correlation coefficient (ICC) was calculated. Results: 155 teeth were analyzed. The ICC was the lowest in 2D, followed by overall, transversal and sagittal 3D. Malmgren scores were systematically higher in 2D, which overestimated RR, especially in the transversal plane on all incisors and canines and in the sagittal plane on the maxillary incisors. 2D respectively leads to 28.0–34.8% of false positives and negatives when discriminating between RR or not. The early stages of RR are often misdiagnosed in 2D, while later stages are more accurate. Conclusions: The original Malmgren index is not suited for 3D images, especially axial, where using dichotomized values (resorption yes/no) leads to overestimation of RR. A low-dose CBCT of the upper incisors could detect RR with high diagnostic accuracy in the early stages of orthodontic treatment, especially in patients with dental trauma or familial RR history.

## 1. Introduction

Root resorption is due to non-bacterial destruction of the mineralized cementum by clastic cells and can be classified as internal or external. External root resorption begins in the cementum and/or dentin and progresses inwards towards the dental pulp. So far, eight types of external root resorption have been described: “surface”, “inflammatory”, “replacement”, “invasive”, “pressure”, “orthodontic”, “physiologic” and “idiopathic” [1]. External root resorption seems to be multifactorial, and several etiologic factors have been suggested in the literature, such as age, sex, genetic predisposition, and tooth and root morphology. Orthodontic root resorption is one of the most important complications of orthodontic treatment [1,2] and has been linked with additional factors, such as the severity of malocclusion, the degree of orthodontic force and treatment duration. Since root resorption is a progressive process, its early and accurate detection during orthodontic treatment is crucial [3].

Root resorption can be assessed by two different methods: directly measuring root length or sorting the degree of root resorption into different levels [4]. Since direct measurements on 2D images can lead to bias due to distortion or magnification, methods categorizing root resorption in degrees or stages are more often found in the literature [5,6]. A widely used index for detecting the degree of horizontal apical root resorption on 2D images is the one published by Malmgren and Levander in 1988 [7]. The Malmgren index grades horizontal apical root resorption from 1 (irregular root contour) to 4 (root resorption exceeding 1/3 of the original root length) [7] and was originally developed to investigate the risk of severe external apical root resorption (EARR) in relation to the apical root form after the first 6–9 months of orthodontic treatment with fixed appliances. Since panoramic radiographs are commonly used in orthodontics, this index is easy to apply in daily practice. It sets a threshold considered clinically relevant and does not depend on standardization of initial radiographs, contrary to linear measurement techniques [4]. In 2017, Alamadi et al. published a modification of the Malmgren index that not only takes horizontal root resorption into account, but also addresses slanted root resorption [5]. Another available index is that of Brezniak and Wasserstein, which describes three degrees of root resorption induced by orthodontic force. Heithersay et al. also suggested four levels of cervical resorption, and Patel et al. proposed a classification for external cervical resorption [6].

The incidence of root resorption varies greatly in literature, in particular depending on the diagnostic technique used [8]. The retrospective study of Bayir et al. reports the incidence of root resorption in any degree to be 27.7%, measured on panoramic radiographs of 1356 orthodontically treated patients. Degree 2 and Degree 3 resorption were reported to be 14.8% combined [8].

Contrary to 2D images, CBCT perceives the exact nature, location and extent of the root resorption. Nevertheless, CBCT is not often used in orthodontic diagnosis and follow up because of the increased radiation it involves compared to other radiologic modalities. This is especially important regarding the fact that the orthodontic population often involves growing patients [5,9,10,11,12,13]. However, studies show that panoramic radiographs tend to overestimate apical root resorption by 20% compared to periapical radiography and that periapical radiographs underestimate apical root resorption compared to 3D radiography [9,10]. Despite these limitations, the Malmgren index is still commonly used in orthodontic research, even on 3D images [5,14,15,16], and there is a lack of literature investigating the diagnostic efficacy of this index in 3D images. In particular, the variation of the diagnostic performance between 2D and 3D according to the severity of root resorption or the position of the teeth remains poorly studied.

The primary aim of this pilot study is to compare the performance (mean differences in the different planes of space, reproducibility, sensitivity and specificity) of the Malmgren index on 2D and 3D radiographs. Additionally, differences per tooth type (central incisors, lateral incisors and canines) will be further explored as a secondary aim. The null hypothesis is that there are no differences in performance of the Malmgren index between 2D and 3D.

## 2. Materials and Methods

### 2.1. Sample Selection

The protocol of this retrospective study was defined prior to the start and was approved by the Medical Ethics Committee of KU Leuven University and University Hospitals Leuven, Belgium, with reference number MP015678.

Subjects with at least one 2D panoramic radiograph and one 3D cone beam CT taken at an interval of less than 3 months, presenting root resorption diagnosed in 2D or 3D on (at least one) incisor and/or canine in any of the two radiographs and having at least one appointment at the unit of Orthodontics of University Hospitals Leuven between 2010 and 2020 were retrospectively included. Patients included in another study were excluded. Initially, subjects of all ages were eligible, but teeth with open apices and not erupted were excluded. The images were taken in the context of dental trauma, endodontic problems, orthodontic examination, post-treatment orthodontic check-up or referrals to the maxillofacial surgery department for advice related to extraction of wisdom teeth. Patients with cleft lip and/or palate and patients with craniofacial syndromes, cysts, tumor-impacted teeth and images of poor quality were excluded.

Sample size estimation was performed by the package “ICC. Sample.Size” for R software, based on the following parameters: for a setting with 2 raters, to ensure with 80% probability that the half width of the two-sided 95% confidence interval for the ICC is no more than 0.1, when the anticipated value of the ICC equals 0.7, the required number of patients is 19.

### 2.2. Methods

All panoramic radiographs were taken with a VistaPano S appliance (Dürr Dental, Bietigheim-Bissingen, Germany) and were viewed and analysed with DBSWIN software version 5.17 (Dürr Dental, Bietigheim-Bissingen, Germany). The CBCT images were taken by either a 3D Accuitomo-XYZ Slice View Tomograph (Morita, Kyoto, Japan) or a Planmeca appliance (Planmeca Oy, Helsinki, Finland). The CBCT images were analyzed using IMPAX Volume Viewing 4.0 (Agfa healthcare, Mortsel, Belgium)

The presence or absence and the degree of external apical root resorption was scored with the Malmgren index in both the 2D and 3D sets separately by two independent observers (Hanne Michielsens and Julie Decreus), who were both orthodontists in training, during the same time period. The Malmgren index consists of five grades: 0 (no root resorption), 1 (irregular root contour), 2 (root resorption apically, amounting to less than 2 mm), 3 (root resorption apically, from 2 mm to 1/3 of the original root length), 4 (root resorption exceeding 1/3 of the original root length) [16]. In 2D, one single score was given, while in the 3D images, four scores were given (one for each plane: axial (3D-A), transversal (3D-T), sagittal (3D-S)) and an overall score (3D-O), by using the mean values of the transversal and sagittal scores. In the axial plane, root resorption was reduced to a binary score: 0 (no root resorption) or 1 (root resorption). Both observers were trained to use the Malmgren index by an experienced observer (Maria Cadenas) and were calibrated prior to the start. Each radiograph (2D and 3D) was scored twice by both observers. Inter- and intra-observer reliability was calculated with the intra-class correlation coefficient (ICC).

### 2.3. Statistical Analysis

In order to investigate measurement reliability, the intraclass correlation coefficient (ICC) was determined with SAS macro icc9, correcting for systemic differences between teeth, and was presented with 95% confidence intervals.

The 2D and 3D datasets were compared on the basis of all available observations (both observers, both ratings). A linear mixed model was used for data analysis with ‘root resorption score’ as the ordinal outcome measurement and ‘type of measurement’ (2D versus 3D) as the explanatory factor. Random intercepts were modelled to deal with clustered data for subject, tooth within subject, and observer. Results are reported as mean difference between both rating types with 95% confidence intervals. Normality of the residuals was checked graphically.

Afterwards, the sensitivity and specificity were estimated with 95% confidence intervals for the 2D score with respect to the different 3D scores as golden standard. Different cut-off values were considered for the ordinal scores (0 vs. 1–4, 0–1 vs. 2–4, 0–2 vs. 3–4, 0–3 vs. 4). All tests were performed at a two-sided 5% significance level. Analyses were performed using SAS software (version 9.4 of the SAS System for Windows) and Figures were constructed using GraphPad Prism 7 (Dotmatics, Boston, MA, USA).

## 3. Results

A total of 20 patients (14 females, 6 males; mean age 17.75 years, 15–22 years) presenting apical root resorption on (at least) one incisor and/or canine were included and analyzed in this study. From the 240 teeth subjected to analysis (all upper and lower incisors and canines), 85 could not be evaluated due to poor quality of the images or presence of an open apex, and were excluded. A final sample of 155 teeth were analyzed in both 2D and 3D, distributed as follows according to tooth type: central upper incisors (CI: 20.26%), lateral uppers incisors (LI: 17.1%), upper canines (C: 17.1%), lower central incisors (CI: 15.2%), lower lateral incisors (LI: 15.2%) and lower canines (C: 15.2%).

### 3.1. Malmgren Index in 3D vs. 2D

**Figure 1:**
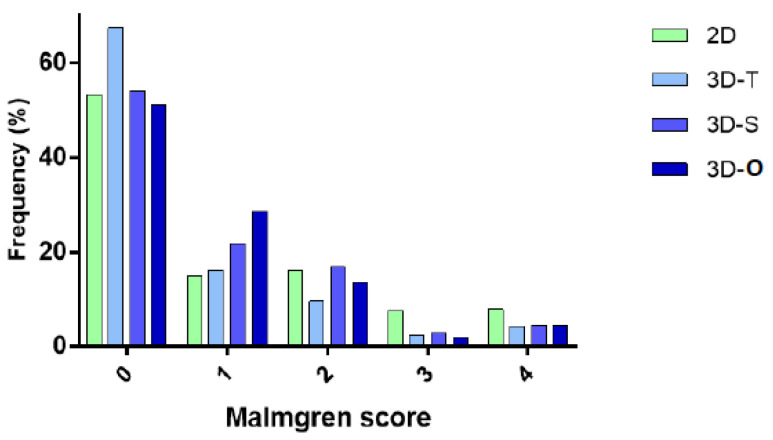
Frequency of Malmgren score in 2D and 3D. Abbreviations: T: transversal plane, S: sagittal plane, O: overall score.

Analysis of the distribution of Malmgren scores in 2D, as well as in the 3D transversal, sagittal and overall scores was normal and show two opposing trends. The percentages of scores equal to 0 or 1 in 2D are similar or lower than those given in 3D, which was considered as the gold standard. On the other hand, the percentages of Malmgren scores given in 2D and representing a Malmgren score of 3 or 4 are almost twice as frequent as the same score in 3D (Figure 1). This suggests an overestimation of the Malmgren score in 2D compared to 3D.

This result is confirmed by the mean difference between both rating types. Significantly lower overall Malmgren scores were given in 3D compared to 2D (*p* < 0.0001, Table 1) in the transversal and sagittal dimensions, with an average difference of −0.425 and −0.199, respectively, which means that 2D systematically overestimates root resorption (Table 1). However, in the axial dimension, a higher probability of obtaining higher scores with 3D was observed.

### 3.2. ICC

**Figure 2:**
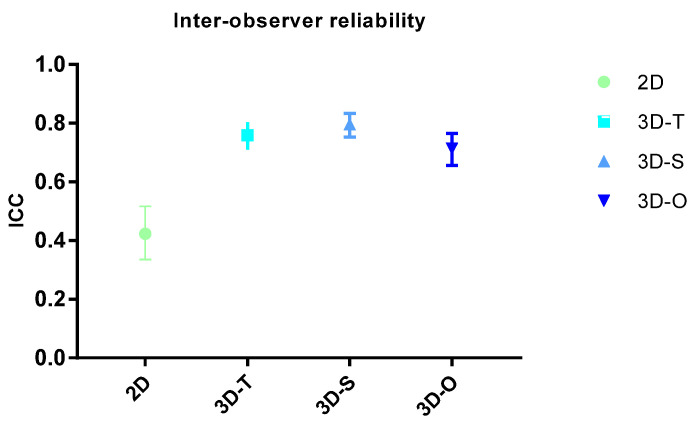
Inter-observer reliability. Abbreviations: O: overall score, S: sagittal plane, T: transversal plane.

The reproducibility of results is an important quality for a diagnostic test. In our study, the Malmgren scores were scored twice by two observers with a one month difference between both measurements. Intra-observer reliability was 0.756 for 2D, 0.886 for 3D-T, 0.898 for 3D-S and 0.827 for 3D overall. The inter-observer reliability was significantly better in 3D than in 2D since the intraclass correlation coefficient (ICC) was higher in 3D (0.7–0.8) than in 2D (0.4). The ICC was the lowest in 2D, followed by overall 3D, transversal and sagittal 3D. Inter-observer reliability was 0.423 for 2D, 0.759 for 3D-T, 0.796 for 3D-S and 0.713 for 3D overall. (Figure 2)

### 3.3. Analysis per Tooth

**Figure 3:**
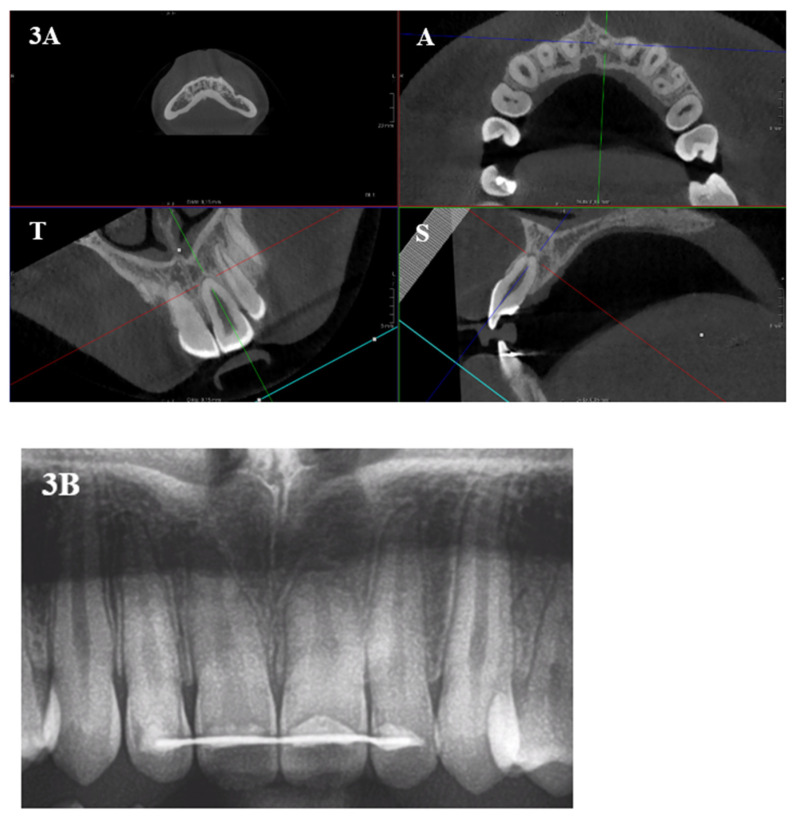
(**3A**) CBCT image of element 21 (A: axial, T: transversal and S: sagittal plane) showing a Malmgren score 1 for the transversal and axial plane and score 2 for the sagittal plane. (**3B**) Two-dimensional panoramic radiograph of upper front teeth and canines, with a Malmgren score of 3 for element 21.

The mean Malmgren score differences were further explored per tooth type (Table 2) The 3D scores in the transversal dimension were significantly lower than in 2D on all studied teeth. However, in the sagittal dimension and the overall score, this was only true for central and lateral incisors in the upper jaw. Although non-significant, upper canines and lower central incisors received higher mean Malmgren scores in 3D than in 2D. This means that 2D overestimates root resorption specifically in the transversal plane on all incisors and canines and in the sagittal plane also on the maxillary central and lateral incisors. In the axial plane, however, since the Malmgren scores had to be dichotomized, a significantly higher probability of scoring root resorption was observed in 3D than in 2D on all teeth, except upper canines (Figure 3).

### 3.4. Sensitivity and Specificity

The comparison between two diagnostic tests is generally based in part on the analysis of the specificity and sensitivity of the results from a first test, taking a second test as the reference test. Table 3 reports the sensitivity and specificity of the 2D results, considering 3D in the different 3D planes as the reference test. It can be observed that 2D leads to 28.0–34.8% of false positives and false negatives when it comes to discriminating between 0 (no resorption) and 1–4 (resorption). These percentages improve when higher Malmgren scores are evaluated: false positives and negatives oscillate between 18 and 33.6% when discriminating scores 0–1 from 2–4, and they drop to 4.1–17.4% when discriminating stages 0–2 vs. 3–4 and 0–3 vs. 4. This suggests that the early stages of root resorption are more often misdiagnosed in 2D than the later stages. The more extensive the root resorption, the more accurate the 2D images are. This result is consistent with the distribution of Malmgren scores in 2D and 3D (Figure 1), since scores 0 and 1 were given less frequently in 2D than in 3D, and scores 3 and 4 were given more often in 2D than in 3D.

## 4. Discussion

Panoramic and periapical radiographs are still the most commonly used method to determine root resorption in orthodontics. Periapical radiographs are more suitable to evaluate the periapical area and are actually more accurate than panoramic radiographs when it comes to evaluating root resorption, especially of the upper incisors due to superimposition of the cervical spine. Additionally, periapical radiographs imply a lower radiation dose [16]. However, they present a very limited field of view, which means that several images need to be taken for a comprehensive evaluation of all potentially affected teeth. Periapical radiographs are also subjected to distortion due to possible positioning errors [3]. As a result of this, panoramic radiographs are more often used to evaluate root resorption during orthodontic treatment. However, they present limitations, such as distortion errors, superimposition of anatomical structures or blurring [5,11,16,17,18,19]. Furthermore, during orthodontic treatment, incisor angulation might change, which can affect the radiographic measurements of tooth length, which means that the amount of root resorption is not evaluated precisely. Finally, the lack of reproducibility is also an important factor that limits the diagnostic accuracy of the panoramic X-ray. The distance between the X-ray source and the film or imaging plate can lead to distortion of panoramic radiographs, due to a difference between the axis motion track of the X-ray appliance and the shape of the inspected parts. This distortion rate may differ depending on the instruments used, the shooting position and the measurement methods. The form and symmetry of the dental arch, the arrangement of the teeth in the arch, tooth shape and angulation, and the surrounding tissues also exert an influence on the image. Tong et al. found the vertical panoramic X-ray magnification rate to be about 25%, which is the same as the magnification rates of X-ray machines [20].

CBCT images allow for direct linear measurements, quantification of root volume and surface area, and superimposition of the same tooth by using two subsequent 3D images. Nevertheless, root resorption is often evaluated on 3D images by using methods originally developed for 2D. Our study shows that using the Malmgren index on the axial dimension of a 3D image is not possible, and using a binary score (resorption: yes or no) yields a higher probability of scoring resorption. When using the 2D Malmgren index in panoramic radiographs, significantly higher scores were given in 2D compared to 3D (*p* < 0.0001, Table 1) in the transversal and sagittal dimensions, which means that 2D systematically overestimates root resorption, which has been confirmed by previous studies [5,18,20,21]. The null hypothesis of the present study is therefore discarded.

Literature reports have shown that upper central and lateral incisors are more prone to orthodontically induced root resorption, especially when preceded by dental trauma, which also happens more often on these teeth [22,23,24]. In the present study, the overestimation of root resorption in 2D was especially evident in the transversal plane on both incisors and canines, and in the sagittal plane only on the maxillary central and lateral incisors. In the axial plane, however, a significantly higher probability of scoring root resorption was observed in 3D than in 2D on all teeth, except upper canines.

The distribution of the Malmgren scores revealed that scores 0 and 1 were given less frequently in 2D than in 3D, and scores 3 and 4 were given more often in 2D than in 3D. The results of specificity and sensitivity showed that the higher the degree of resorption, the higher the sensitivity and specificity of 2D gets, or in other words, the lower the risk for false positives and negatives. Together, these findings suggest that the early stages of root resorption are more often misdiagnosed in 2D than the later stages, and that the more extensive the root resorption, the more accurate the 2D diagnosis is. Finally, the inter-observer reliability is significantly better in 3D than in 2D, which decreases the risk of two practitioners establishing different diagnoses on the same image.

The ‘ALARA’ principles dictate that caution should be taken to minimize radiation exposure to patients, which should be as low as reasonably achievable [5]. However, research proposes to move from ‘ALARA’ towards ‘ALADAIP’ (as low as diagnostically acceptable, being indication-oriented and patient-specific) [20,21]. Regarding root resorption, the literature shows that 2D radiography cannot supply the needed diagnostic information [17,18]. On the other hand, radiation exposure could be controlled with limited field CBCT-generated volumes, which can provide a detailed 3D image, and every new generation of hardware and software is adapted to minimize the radiation dose [3,25,26].

If apical external root resorption is found in the first 6–12 months of orthodontic treatment, the risk for further root resorption is considered to be high [4]. After detection of EARR, the literature suggests either reducing the degree of orthodontic force, spacing appointments or pausing treatment, depending on the extent of the root resorption [4]. If root resorption is detected early in treatment, the treatment plan can be adapted accordingly [4,16]. Taking these guidelines into consideration, it could be diagnostically interesting to take a CBCT with a small field of view (FOV) and large voxel size on the maxillary incisors 6 to 9 months into orthodontic treatment to accurately detect root resorption on those teeth more prone to it, while minimizing the radiation dose. An OPG has an effective dose varying from 0.004 to 0.03 mSv, while a CBCT taken with a large/medium FOV has an effective dose of 0.07–0.55 mSv, and a CBCT with a small FOV has an effective dose of 0.005–0.5 mSv. Taking a CBCT with a small FOV could become a standard procedure in patients with thin roots, current presentation of root resorption, a familial history of root resorption or personal history of trauma to the front teeth [24].

The efficacy of low-dose CBCT has been proven in the literature. Yeung et al. evaluated the image quality of scans taken for endodontic indications and found that low-dose CBCT did not negatively affect the perception of image quality and could therefore be used for diagnostic purposes prior to or following endodontic treatment [27]. Ruetters et al. showed that low-dose CBCT is a precise and reliable method for detecting and measuring furcation defects in mandibular and maxillary molars in an experimental setting. They reported that low-dose CBCT has the potential to improve treatment planning and treatment monitoring, involving a far lower radiation dose than conventional high-dose-CBCT [28]. Oenning et al. (DIMITRA research) demonstrated that it is possible to achieve a good balance between dose and image quality using a small FOV, and even small details can be assessed in likely optimized protocols (e.g., evaluation of lamina dura and periodontal ligament space). However, it is worth mentioning that greater FOV restrictions (e.g., 5 × 5 cm) must be used for some specific indications favoring both dose reduction and imaging quality improvement [25].

Our results suggest a second possibility for reconciling diagnostic efficacy and radiation protection. Since 2D radiography tends to overestimate root resorption in the transverse and sagittal planes, panoramic radiographs could remain the diagnostic screening method, but a positive result (meaning a non-zero resorption score) should be confirmed by CBCT. According to our results, this approach would, however, underestimate the scores in the axial dimension.

Lastly, it is important to take in consideration the limitations of the present study. For example, it could have been interesting to investigate the potential reasons for the root resorption observed in the included patients, or the type and length of their orthodontic treatment, as well as the time between the end of treatment and the radiographic images. A larger sample size could also allow for more conclusive results and for stratification of patients according to age. Finally, future research could compare evaluation of root resorption before and after orthodontic treatment in 3D with the scores given in 2D by other, more traditional methods, such as using the Malmgren index or lineal measurements on periapical radiographs.

## 5. Conclusions

This pilot study suggests that the Malmgren index cannot be used in 3D, since significantly lower overall Malmgren scores were given in 3D compared to 2D (*p* < 0.0001) in the transversal and sagittal dimensions. 2D systematically overestimates root resorption, especially in the transversal plane on all incisors and canines, and in the sagittal plane on the maxillary central and lateral incisors. 2D leads to 28.0–34.8% of false positives and negatives when it comes to discriminate between no resorption vs. resorption. These percentages improve when higher Malmgren scores are evaluated, which means that the early stages of root resorption are more often misdiagnosed in 2D than the later stages, where 2D diagnosis is more accurate. Conclusions should, however, be extracted with caution due to the limited sample size of our study. An adaptation of the Malmgren index for 3D images is needed, especially on the axial dimension, where using dichotomized values (resorption yes/no) leads to overestimation of root resorption. Taking a low-dose CBCT with a small field of view focused on the upper incisors could help detect root resorption with high diagnostic accuracy in the early stages of orthodontic treatment, especially in patients with prior dental trauma or familial history of root resorption.

## Figures and Tables

**Table 1:** Comparison between global Malmgren score in 3D vs. 2D.

**Comparison**	**Mean Difference (95% CI)**	***p*-Value**
3D-T vs. 2D	−0.425 (−0.502; −0.348)	<0.0001
3D-S vs. 2D	−0.199 (−0.277; −0.120)	<0.0001
3D-O vs. 2D	−0.226 (−0.303; −0.149)	<0.0001
**Comparison**	**Odds Ratio (95% CI)**	***p*-Value**
3D-A vs. 2D	0.168 (0.113; 0.250)	<0.0001

Abbreviations: T: transversal plane, S: sagittal plane, O: overall score, A: axial plane, CI: confidence interval. Note that negative mean differences refer to lower Malmgren scores in 3D. Odds ratio > 1 refer to a higher probability of root resorption in 3D compared to 2D.

**Table 2:** Comparison between Malmgren score in 3D vs. 2D per tooth.

	Mean Score Differences (*p*-Value)					
	Upper Jaw			Lower Jaw		
	CI	LI	C	CI	LI	C
**3D-T vs. 2D**	−0.570 (<0.0001) *	−0.546 (<0.0001) *	−0.121 (0.0441) *	−0.593 (<0.0001) *	−0.396 (0.0001) *	−0.260 (0.0012) *
**3D-S vs. 2D**	−0.648 (<0.0001) *	−0.204 (0.0307) *	0.022 (0.7283)	0.046 (0.7095)	−0.125 (0.2410)	−0.115 (0.1676)
**3D-O vs. 2D**	−0.484 (<0.0001) *	−0.288 (0.0032) *	−0.013 (0.8424)	−0.172 (0.1361)	−0.187 (0.0715)	−0.102 (0.2165)
**3D-A vs. 2D**	**Odds Ratio (*p*-Value)**					
	**Upper Jaw**			**Lower Jaw**		
	**CI**	**LI**	**C**	**CI**	**LI**	**C**
	0.096 (<0.0001) *	0.750 (0.0032) *	0.9546 (0.8424)	0.390 (0.0392) *	0.112 (0.0004) *	0.176 (0.0303) *

Abbreviations: A: axial plane, C: canine, CI: central incisor, LI: Lateral Incisor, O: overall score, S: sagittal plane, T: transversal plane. Note that negative mean differences refer to lower Malmgren scores in 3D and Odds ratio >1 refer to a higher probability of root resorption in 3D than in 2D. * significant values (*p* < 0.05).

**Table 3:** Sensitivity and specificity of 2D compared to 3D in the different 3D planes.

Malmgren Scores			3D-T	3D-S	3D-O	3D-A
**0 vs. 1–4**	**% Sensitivity (CI)**	True positives	72.0 (65.2; 78.1)	66.4 (60.6; 71.9)	65.8 (60.1; 71.1)	NA
		False negatives	28.0 (21.9; 34.8)	33.6 (28.1; 39.4)	34.2 (28.9; 39.9)	NA
	**% Specificity (CI)**	True negatives	65.2 (60.4; 69.8)	69.7 (64.4; 74.6)	70.8 (65.4; 75.8)	NA
		False positives	34.8 (30.2; 39.4)	30.3 (25.4; 35.6)	29.2 (24.2; 34.6)	NA
**0–1 vs. 2–4**	**% Sensitivity (CI)**	True positives	82.0 (73.1; 89.0)	66.4 (58.3; 74.0)	75.6 (67.0; 82.9)	79.8 (69.9; 87.6)
		False negatives	18.0 (11.0; 26.9)	33.6 (26.0; 41.7)	24.4 (17.1; 33.0)	20.2 (12.4; 30.1)
	**% Specificity (CI)**	True negatives	78.0 (74.2; 81.5)	79.3 (75.3; 82.9)	79.2 (75.3; 82.7)	76.4 (72.5; 80.0)
		False positives	22.0 (18.5; 25.8)	20.7 (17.1; 24.7)	20.8 (17.3; 24.7)	23.6 (20.0; 27.5)
**0–2 vs. 3–4**	**% Sensitivity (CI)**	True positives	90.2 (76.9; 97.3)	82.6 (68.6; 92.2)	92.5 (79.6; 98.4)	NA
		False negatives	9.8 (2.7; 23.1)	17.4 (7.8; 31.4)	7.5 (1.6; 20.4)	NA
	**% Specificity (CI)**	True negatives	89.7 (86.9; 92.1)	89.8 (87.0; 92.1)	89.7 (86.9; 92.1)	NA
		False positives	10.3 (7.9; 13.8)	10.2 (7.9; 13.0)	10.3 (7.9; 13.1)	NA
**0–3 vs. 4**	**% Sensitivity (CI)**	True positives	88.5 (69.8; 97.6)	89.3 (71.8; 97.7)	89.3 (71.8; 97.7)	NA
		False negatives	11.5 (2.4; 30.2)	10.7 (2.3; 28.2)	10.7 (2.3; 28.2)	NA
	**% Specificity (CI)**	True negatives	95.6 (93.6; 97.1)	95.9 (94.0; 97.4)	95.9 (94.0; 97.4)	NA
		False positives	4.4 (2.9–6.4)	4.1 (2.6; 6.0)	4.1 (2.6; 6.0)	NA

Abbreviations: CI: Confidence Interval. Note that true positives refer to the % of 3D scores indicating root resorption that also indicate the same in 2D, and false negatives refer to the % of 3D scores indicating root resorption that are scored as no root resorption on 2D. True negatives are the % of 3D scores indicating no root resorption that also indicate the same on 2D, and false positives are the % of 3D scores indicating no root resorption that indicate root resorption on 2D.

## Data Availability

Data is contained within the article.
